# Histaminergic regulation of food intake

**DOI:** 10.3389/fendo.2023.1202089

**Published:** 2023-06-27

**Authors:** Axelle Khouma, Moein Minbashi Moeini, Julie Plamondon, Denis Richard, Alexandre Caron, Natalie Jane Michael

**Affiliations:** ^1^ Institut Universitaire de Cardiologie et de Pneumologie de Québec, Quebec, QC, Canada; ^2^ Faculté de Pharmacie, Université Laval, Québec, QC, Canada; ^3^ Faculté de Medicine, Université Laval, Québec, QC, Canada; ^4^ Montreal Diabetes Research Center, Montreal, QC, Canada

**Keywords:** histamine, food intake, hypothalamus, neurometabolism, melanocortin, leptin, histamine receptors, GPCR

## Abstract

Histamine is a biogenic amine that acts as a neuromodulator within the brain. In the hypothalamus, histaminergic signaling contributes to the regulation of numerous physiological and homeostatic processes, including the regulation of energy balance. Histaminergic neurons project extensively throughout the hypothalamus and two histamine receptors (H1R, H3R) are strongly expressed in key hypothalamic nuclei known to regulate energy homeostasis, including the paraventricular (PVH), ventromedial (VMH), dorsomedial (DMH), and arcuate (ARC) nuclei. The activation of different histamine receptors is associated with differential effects on neuronal activity, mediated by their different G protein-coupling. Consequently, activation of H1R has opposing effects on food intake to that of H3R: H1R activation suppresses food intake, while H3R activation mediates an orexigenic response. The central histaminergic system has been implicated in atypical antipsychotic-induced weight gain and has been proposed as a potential therapeutic target for the treatment of obesity. It has also been demonstrated to interact with other major regulators of energy homeostasis, including the central melanocortin system and the adipose-derived hormone leptin. However, the exact mechanisms by which the histaminergic system contributes to the modification of these satiety signals remain underexplored. The present review focuses on recent advances in our understanding of the central histaminergic system’s role in regulating feeding and highlights unanswered questions remaining in our knowledge of the functionality of this system.

## Introduction

Histamine is a small biological molecule (biogenic amine) that is widely distributed throughout the body. Although probably best recognized for its importance in arousal regulation and allergic inflammatory reactions, histamine plays a role in a diverse range of biological functions. This includes the regulation of energy balance, sleep and wakefulness, thermoregulation, gastrointestinal function, immune responses, and learning and memory (1–3). Within the central nervous system (CNS), a population of neurons located in the posterior hypothalamus provide the sole source of neuronal histamine to the brain (4–6) and can be identified based on the expression of histidine decarboxylase (HDC), the enzyme required for histamine synthesis (7, 8). These histaminergic neurons project extensively throughout the CNS, and strongly innervate multiple hypothalamic nuclei known to influence energy homeostasis and feeding behaviors (9–11). While histamine is known to impact food intake via its actions in the hypothalamus (12, 13), the precise mechanisms by which it does so are still being uncovered. The present review focuses on recent advances in understanding of the central histaminergic system’s role in regulating food intake, including potential interactions with satiety signals and neuropeptide/neurotransmitter systems implicated in energy balance regulation.

## Histaminergic neurons

**Figure 1:**
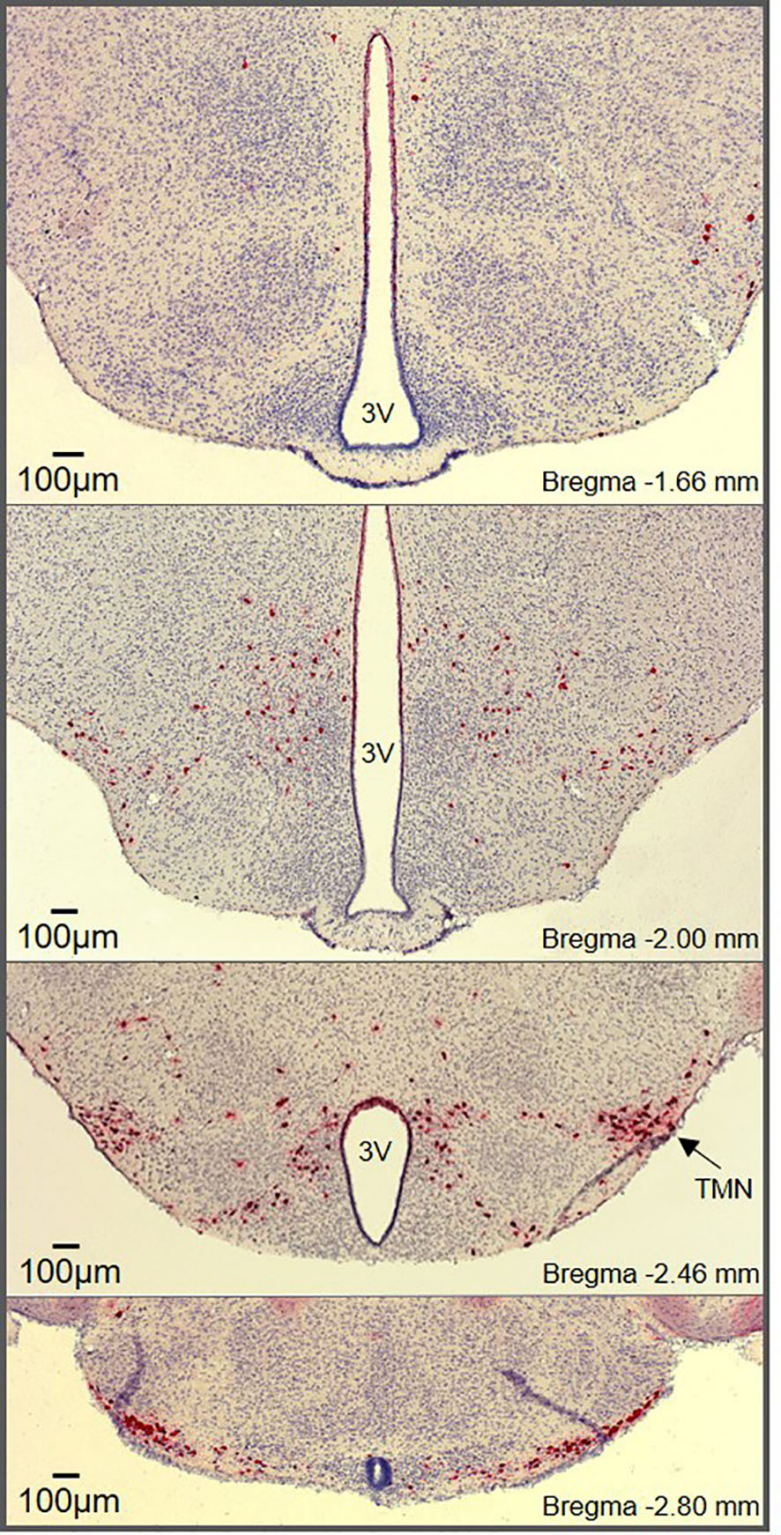
Histaminergic neuron distribution throughout the hypothalamus. RNAscope^®^ *in situ* hybridization (ISH) targeting *Hdc* shows histaminergic neurons (red) densely packed in the core region of the tuberomammillary nucleus (marked TMN) along with diffusely scattered histaminergic neurons throughout the hypothalamus. The RNAscope^®^ ISH was performed on hypothalamic brain slices (25µm) from male mice according to the manufacturer’s instructions (Advanced Cell Diagnostics, Inc., USA).

Histaminergic neuron somas are confined to the tuberomammillary nucleus (TMN) in the posterior hypothalamus (**Figure 1**) but have widespread projections that extensively innervate the CNS. This includes major brain regions including the cortex, brainstem, hippocampus, striatum, nucleus accumbens, amygdala, and substantia nigra, as well as multiple intrahypothalamic projections (9–11, 14). The diffuse projection patterns correlate with the multiple functions associated with histaminergic neurons, which have been comprehensively reviewed elsewhere (1, 3, 15). In contrast to the diffuse and well characterized projections of histaminergic neurons, difficulties occurred with initial attempts to identify afferent inputs to the histaminergic neurons, likely due to the inherent limitations of retrograde tracing studies, including potential spread to surrounding tissue and labeling of fibers of passage (16, 17). However, significant afferent input to the histaminergic neurons has since been identified, with inputs originating from the ventrolateral preoptic area (VLPO) (17–19) and the lateral hypothalamus (20–23). Importantly, the TMN can be subdivided into 3-5 different subregions depending on the classification method used (9, 24, 25). While histaminergic neurons are usually acknowledged to reside in the ‘TMN core’, their distribution within the hypothalamus (including the dorsal and bridge regions of the TMN) is much more widespread than typically appreciated (**Figure 1**), with some degree of variability observed between species (26, 27). However, the anatomical location of the histaminergic neurons and distribution of fibers throughout the brain appears comparable in humans to that described in rodents (28).

The location of the histaminergic neurons within the hypothalamus raises the potential for their involvement in the regulation of feeding. Many of the histaminergic neurons lay in close proximity to the third ventricle or are located on the ventral surface of the brain, suggesting, like other hypothalamic neurons, a potential for the detection of circulating hormones and neuropeptides (29). Moreover, histaminergic neuron fibers densely innervate the hypothalamus, including key hypothalamic nuclei known to regulate energy balance. While early studies required colchicine treatment to visualize histamine containing neurons (4), targeting of HDC (the enzyme required for histamine synthesis) allowed for the identification of dense fiber networks throughout the hypothalamus (6). Studies examining HDC immunoreactivity alone or in combination with paired retrograde tracer studies reveal high to very high density of histaminergic fibers in hypothalamic regions that regulate energy homeostasis. This includes the paraventricular nucleus of the hypothalamus (PVH), ventromedial hypothalamus (VMH), lateral hypothalamus (LH), dorsomedial hypothalamus (DMH) and the arcuate nucleus (ARC) (6, 9, 10). Use of newer immunohistochemical methods, with increased sensitivity for the visualization of histamine immunoreactive fibers and terminals, provided additional support for a moderate density of histaminergic fibers in the PVH, VMH, DMH, LH, and ARC (11). While identification of fiber tracts does not necessarily indicate functional connections, the presence of histamine receptors in these regions supports the role of histamine in regulating the activity of key metabolic neurons located in these areas of the hypothalamus.

## Histamine receptor signaling

Histamine exerts its pleiotropic effects by binding to four subtypes of histamine receptors (HR), three of which are located within the brain (H1R, H2R, and H3R) (30–33). HRs belong to the family of G protein–coupled receptors (GPCRs), which interact with G proteins located in the plasma membrane. When a ligand binds to a GPCR, it causes a conformational change that triggers the interaction between the GPCR and nearby heterotrimeric G proteins. This promotes the exchange of a GDP for a GTP on the Gα subunit, resulting in its dissociation from Gβγ (34). There are four main families of Gα subunits: Gαi, Gαq, Gαs, and Gα12 (35). Gα subunits and Gβγ can activate different signaling pathways.

**Figure 2:**
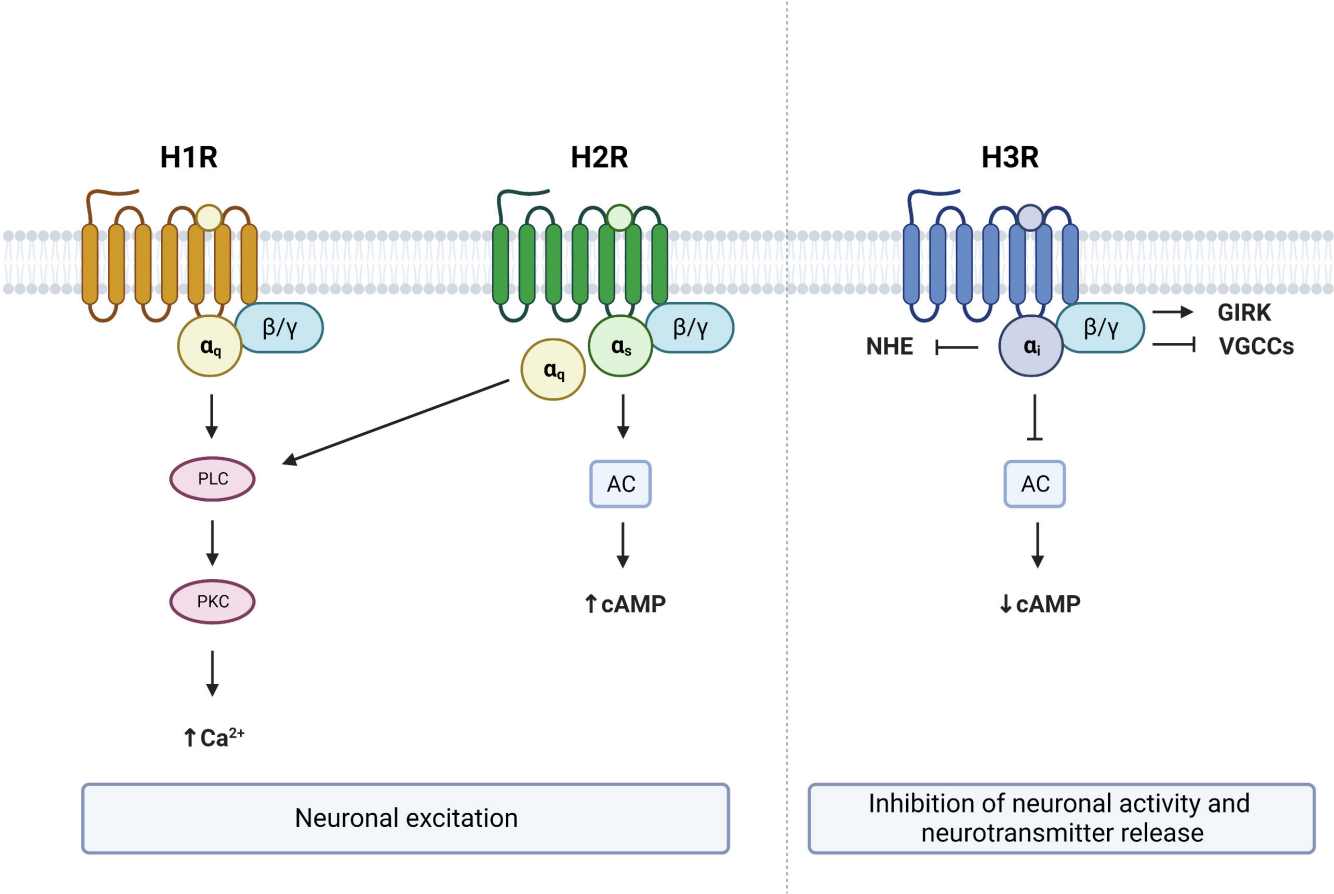
G protein–coupled receptor (GPCR) signaling from brain expressed histamine receptors. Histamine activation of H1R and H2R lead to neuronal excitation via Gαq and/or Gαs dependent mechanisms respectively. Activation of H3R leads to neuronal inhibition and suppression of neurotransmitter release. PLC, phospholipase C; PKC, protein kinase C; AC, adenylate cyclase; NHE, sodium–proton exchanger; GIRK, G protein-gated inwardly rectifying potassium channels; VGCCs, voltage-gated calcium channels; cAMP, cyclic adenosine monophosphate. Figure created with BioRender.com.

Identified in 1966, the H1R subclass of histamine receptors (gene symbol: HRH1) primarily couples to Gαq, resulting in the activation of the phospholipase C (PLC) signaling pathway (36–40) (**Figure 2**). This leads to the subsequent cleavage of phosphatidylinositol 4,5-bisphosphate (PIP2) into diacyl glycerol (DAG) and inositol 1,4,5-trisphosphate (IP3). These second messengers respectively activate protein kinase C (PKC) and promote the mobilization of Ca^2+^ (41). Accumulation of IP3, DAG and Ca^2+^ following histamine was shown to be prevented with the H1R inverse antagonist pyrilamine (42–45), while the H1R inverse agonist chlorpheniramine was reported to block the stimulatory effect of histamine on PLC and Ca^2+^ (46), confirming the involvement of H1R in the Gαq-dependent actions of histamine. One report also suggests that activation of H1R by histamine increases cAMP levels through Gβγ, an effect that is prevented by the H1R inverse agonist pyrilamine (47). As such, H1R activation and stimulation of it signaling cascade has excitatory effects and is associated with membrane depolarization in neurons (48–52).

The H2R subclass of histamine receptors (gene symbol: HRH2), often referred to as the histamine gastric receptor, couples to both Gαq and Gαs proteins (39, 53). As a consequence, histamine binding to H2R stimulates both PLC and adenylate cyclase (AC) through Gαq and Gαs proteins respectively (53) (**Figure 2**). Activation of Gαs proteins in turn increases cAMP, an effect that is prevented by the HRH2 antagonist lafutidine (54). Increased cytosolic cAMP then leads to the activation of protein kinase A (PKA), which has been shown to stimulate neurons (55). Therefore, histamine binding to H2Rs also has excitatory actions within the brain, and results in depolarization of neurons through increased Ca^2+^, cAMP and PKA (56, 57).

The H3R subclass of histamine receptors (gene symbol: HRH3, previously known as GPCR97) primarily couples to Gαi proteins (39, 58) and functions as an inhibitory auto- or hetero-receptor in the brain (59–62). Activation of H3R results in AC inhibition and a subsequent reduction of cAMP levels (63, 64) (**Figure 2**). In contrast to the excitatory effects of H1R and H2R, binding of the H3R by histamine results in a suppression of neuronal activity and inhibition of neurotransmitter release (15, 60, 65, 66). Several mechanisms can contribute to the inhibitory effects of H3R. First, the Gβγ subunit of Gαi-coupled receptors has been shown to activate G protein-gated inwardly rectifying potassium (GIRK) channels (66, 67). Second, H3R activation can reduce neurotransmitter release by inhibiting N- and P/Q-type voltage-gated calcium channels again through the Gβγ subunit (68, 69). Third, H3R activation has been shown to reduce the activity of the sodium–proton exchanger (NHE), which is under the control of Gαi (70, 71). Therefore, activation of H3R has opposite effects on neuronal activity to that of H1R or H2R activation.

## Central histaminergic system and the regulation of feeding

### Histamine synthesis

The central histaminergic system has been implicated in the regulation of food intake through multiple different strategies used to manipulate the system. This includes altering the body’s ability to produce histamine. Genetic knock-out of histidine decarboxylase (HDC-KO) has been used to prevent the synthesis of histamine, resulting in histamine deficient mice. Studies using these mice suggest that HDC-KO animals are more susceptible to develop obesity as they age, or after consumption of a high fat diet (72–74). Detailed analyses of food intake in these mice are lacking, however, one study suggests that HDC-KO mice are not hyperphagic, but have an increased feed efficiency (72). However, the increased body weight in HDC-KO mice could be confounded by the decreased locomotor activity observed in these animals (75–77). An alternate method to deplete histamine is the use of α-Fluromethyl-[S]-histidine (α-FMH) which is a suicide inhibitor of histamine synthesis. Chemical inhibition of histamine synthesis with α-FMH has consistently been associated with an increase in food intake (78–82), suggesting that overall, histamine may be anorexigenic. However, such genetic or chemical methods preventing histamine synthesis provide limited and unspecific information regarding histamine’s ability to influence feeding, due to a loss of histaminergic tone at all histamine receptors simultaneously.

### H1R activation suppresses food intake

**Figure 3:**
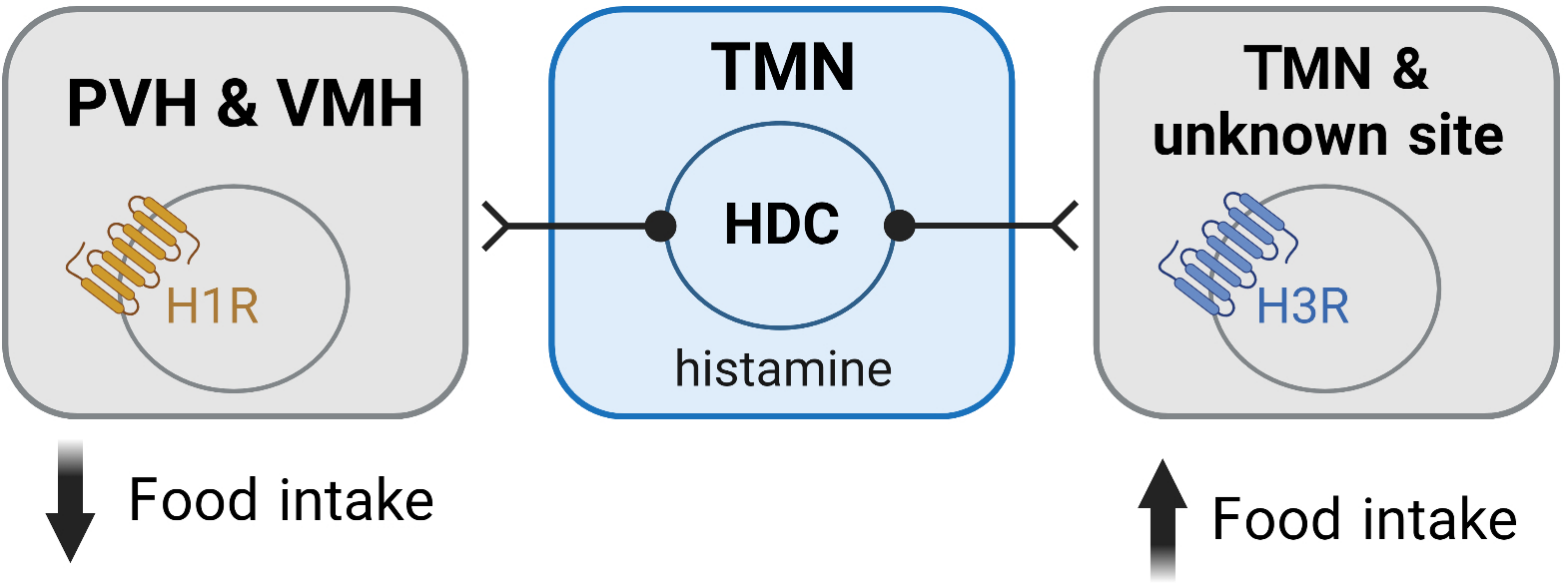
Histamine mediates its effects on feeding via activation of histamine receptors within the hypothalamus. Activation of the H1R is associated with an anorexigenic effect and is believed to be mediated via H1Rs expressed in the PVH and VMH. In contrast, activation of the H3R is orexigenic and occurs via autoinhibition of the histaminergic neurons. H3Rs in unidentified sites may also contribute to the orexigenic effects of H3R activation. Figure created with BioRender.com.

The H1R is generally accepted to mediate the suppression of food intake induced by histamine (**Figure 3**). Early studies demonstrated that intracerebroventricular (ICV) injection of H1R antagonists stimulated feeding (12, 83). Moreover, the effects of pharmacological strategies increasing brain histamine levels, which are associated with a suppression of food intake, are attenuated, or abolished in the presence of H1R antagonists (78, 84). These actions are consistent with the increased food intake and weight gain seen with first-generation antihistamines used to treat allergies (85–87) which are all inverse agonists of the H1R (88).

More recently, global H1R knockout (H1R-KO) mice have been developed (89) and food intake and body weight studies in these mice overwhelmingly backed up that obtained with pharmacological ligands targeting the H1R. The ability of α-FMH to stimulate food intake is lost in H1R-KO mice (79) and histamine’s ability to suppress food intake and body weight is reduced in H1R-KO mice compared to control mice (90). Moreover, the ability of betahistine, which enhances histamine levels and acts as an agonist of H1R, to reduce food intake and body weight is absent in H1R-KO mice (91). Together, these data strongly support the idea that histamine’s actions at the H1R are anorexigenic. Interestingly, H1R-KO mice do not show any changes in food intake or body weight when fed a standard chow diet (90, 92). However, with age or high fat diet feeding, H1R-KO mice accumulate fat mass and develop obesity (90, 92, 93), which is consistent with what was seen in mice completely deficient of histamine (HDC-KO) as discussed above. Additionally, H1R-KO mice display a decreased anorexigenic response to thyrotropin releasing hormone (TRH), neurotensin, nesfatin-1, and estradiol (94–97), suggesting that the H1R may contribute to the suppression of feeding normally induced by these anorexigenic peptides.

While the ability of histamine to suppress feeding is well demonstrated to occur via H1R, the exact neuronal populations and mechanisms responsible for these effects are not well understood. Studies where H1R antagonists were directly infused into different hypothalamic nuclei have demonstrated that blockade of H1R only in the paraventricular (PVH) and ventromedial (VMH) hypothalamus stimulate feeding (12, 13) (**Figure 3**). Similarly, micro infusion of α-FMH to decrease local histamine concentrations, only has effects on food intake when infused in the PVH and VMH (78, 82, 98). While H1R agonists have been shown to induce markers of cell activity (c-Fos) only in the PVH (91), extracellular recording techniques demonstrate that the H1R antagonist chlorpheniramine inhibits neurons in the VMH (83). Despite these studies suggesting that the PVH and VMH are the sites where H1R activation has its anorexigenic effects, future studies are required to further elucidate the mechanisms involved, including the chemical phenotype of the cells in these nuclei mediating the anorexigenic effects of H1R activation.

### H2R activation does not influence food intake

When it comes to central H2Rs, there is limited evidence indicating that they have any role in regulating feeding. Importantly, H2R antagonists administered ICV or directly to hypothalamic regions have no effect on food intake (12, 83, 99, 100). Furthermore, the H2R antagonist ranitidine has been shown to have no effect on histamine-induced suppression of food intake, whereas both H1R and H3R antagonists influenced this effect (84). It should be noted that while H2R antagonists can influence feeding when taken orally or infused directly into the gut, peripheral mechanisms including H2R effects on gastric acid secretion and gut hormones likely contribute to these effects (101–103). Furthermore, it is unsurprising that centrally expressed H2Rs do not influence food intake given that these receptors are most strongly expressed in extrahypothalamic regions such as the cortex, hippocampus, striatum, basal ganglia, and amygdala (30, 33, 104). Together, these data strongly suggest that central H2Rs do not contribute to the homeostatic regulation of food intake.

### H3R activation stimulates food intake

Pharmacological studies indicate that activation of the H3R is orexigenic, with H3R agonist delivery directly to the brain stimulating food intake (105–107) (**Figure 3**). In line with these observations, blockade or inverse agonism of the H3R suppresses food intake (78, 82, 84, 100, 107, 108). The capacity of H3R inverse agonists/antagonists to reduce food intake has also been shown to minimize weight gain occurring in models of diet-induced obesity and to reduce body weight in obese rodents (108–112). Moreover, H3R inverse agonists/antagonists suppress food intake in conditions associated with an increased orexigenic drive, i.e., in the fasted state or following neuropeptide Y (NPY) administration (105, 113). In one study, food intake in rats that received a single dose of thioperamide, a H3R antagonist, was significantly less for two days compared to controls (82). The suppression of food intake induced by H3R antagonists has also been demonstrated to occur in non-rodent species including pigs and non-human primates (114). Together, these studies demonstrate that histamine’s actions at the H3R stimulate feeding, and blockade of this receptor is associated with anorexigenic effects.

The ability of H3R inverse agonists/antagonists to suppress food intake is largely assumed to occur by removing the normal auto-inhibition of histaminergic neurons (**Figure 3**), thereby increasing histamine levels and enhancing action at the anorexigenic H1R (78, 105, 115). However, the H3R also functions as a heteroreceptor and has been shown to be expressed in several brain regions other than the TMN (32, 58, 60, 116). Importantly, the H3R can suppress the release of multiple neurotransmitters including serotonin (117, 118), dopamine (119, 120), noradrenaline (118, 121), acetylcholine (122, 123) and GABA (124–126), neurotransmitters that are implicated in the regulation of feeding. This raises the potential for H3R inverse agonism/antagonism to influence food intake via transmitters other than histamine (**Figure 3**). However, such a possibility has not been comprehensively assessed.

In comparison with pharmacological studies targeting the H3R, experiments using global H3R-KO mice have generated diverging and less consistent findings. H3R-KO mice were shown to consume more food and have an increase in body weight from approximately 10 weeks of age (127). Such findings seem counterintuitive considering that the KO of H3Rs should remove the auto-inhibition of the histaminergic neurons and enhance anorexigenic actions at the H1R. However, it has been demonstrated that H3R-KO mice actually have decreased histamine levels in the hypothalamus and cortex (127, 128) potentially contributing to this effect. In contrast to the food intake effects reported by Takahashi et al. (127), others have suggested that a decrease in food intake occurs in H3R-KO mice, however, food intake was normalized to body weight, making any absolute changes difficult to assess (129). While genetic mouse models can reveal important insights into the mechanistic underpinnings of physiology and behavior, developmental and compensatory actions can occur, especially in relation to fundamental processes such as eating. Moreover, the function of the H3R as a heteroreceptor adds another level of complexity, whereby knockout of H3R could simultaneously influence multiple neurotransmitter systems. Despite some conflicting results obtained in knockout animals, it is clear that the H3R plays an important role in regulating food intake, and its activation is generally orexigenic.

## Histaminergic system and interaction with key metabolic signals

### Leptin

In addition to histamine’s ability to influence feeding, the central histaminergic system has been suggested to interact with other signals reflective of the metabolic state. This includes leptin, a hormone produced by adipose tissue that acts in the CNS to regulate energy metabolism (130). Circulating leptin levels occur in proportion to fat mass and decrease with periods of fasting (131, 132), therefore, acting as a signal of energy reserves to the brain. Exogenous leptin administration is associated with a suppression of food intake, a reduction in body weight, and an upregulation of uncoupling protein 1 (UCP1) expression in adipose tissue depots, all of which have been suggested to require a fully functioning histaminergic system (79, 80, 92, 133). Studies in which histamine synthesis was chemically inactivated failed to observe the normal leptin-induced suppression of food intake and decrease in body weight (79, 80, 133). These effects have been linked to the H1R, as studies performed in mice globally lacking H1R show similar effects. In H1R-KO mice, leptin’s effect on food intake and body weight is suppressed or absent compared to that seen in control animals (79, 92). Additionally, leptin’s ability to decrease body fat percentage and upregulate UCP1 in brown adipose tissue was suppressed in H1R-KO mice (92). Moreover, genetic disruption of histamine synthesis (HDC-KO mice) leads to impairments in leptin sensing and regulation (72, 74, 134). While these studies suggest that the histaminergic system may mediate some of the anorexigenic effects of leptin, the mechanisms by which the histaminergic system regulates the actions of leptin in the CNS remains to be determined. Interestingly, the core region of the TMN, where the histaminergic neurons reside, does not express the long form of the leptin receptor (LepR) (135), which likely precludes direct effects of leptin on the histaminergic neurons themselves. In contrast, LepR is expressed in sub-populations of neurons located in the lateral hypothalamus (136–138) a region known to directly and indirectly influence the histaminergic neurons (139, 140). However, the potential for leptin to influence the activity of histaminergic neurons via presynaptic inputs has not previously been investigated. Future studies are required to determine the sites and mechanisms by which histamine and leptin signaling may converge within the brain.

### Melanocortin system

The central melanocortin system is one of the best-characterized brain circuits regulating food intake and energy expenditure (141–144). Melanocortin peptides, derived from the proopiomelanocortin (POMC) pre-prohormone, form a crucial component of this system and act at cognate melanocortin receptors to influence energy balance (145, 146). Importantly, recent work has identified that histaminergic neurons are sensitive to activation of the melanocortin 4 receptor (MC4R) (147). Using single neuron *ex vivo* electrophysiological recordings from genetically identified histaminergic (HDC) neurons, we demonstrated that approximately 40% of histaminergic neurons are excited by the non-selective MC3R/MC4R agonist melanotan II (MTII) or a selective MC4R agonist (THIQ) (147). These MC4R-mediated effects were shown to modify glutamatergic tone to the histaminergic neurons (147). Moreover, the interaction between the melanocortin and histaminergic systems was shown to be important for feeding regulation. Chemogenetic inhibition of the histaminergic neurons using an inhibitory Designer Receptor Exclusively Activated by Designer Drugs (DREADD) approach (148–150), enhanced the anorexigenic response to central infusion of MTII (147). This study found that melanocortin system activation results in unabated anorexia once the histaminergic neurons are silenced and suggests that, under normal conditions, the melanocortin-dependent activation of histaminergic neurons acts naturally as a negative feedback loop of the anorexigenic effects of the melanocortin system (147). Despite this important observation demonstrating histaminergic neurons are sensitive to key metabolic signals conveyed by the melanocortin system, the downstream mechanisms by which histaminergic neurons restrain the anorexigenic effects of melanocortin system activation remain to be identified.

### Other appetite-related hormones

The ability of other appetite-related hormones to influence the activity and function of the histaminergic neurons has not been intensively investigated. One previous study suggested that ghrelin may activate the histaminergic neurons, as increased c-Fos expression, an indirect marker of cellular activity, was observed in the TMN following central administration of ghrelin (151). However, the receptor for ghrelin, the growth hormone secretagogue receptor (GHSR), is not expressed in the TMN (152) and *Ghsr* mRNA is not detected in transcriptomic profiling of histaminergic neurons (153). This likely prevents any direct post-synaptic modulation of histaminergic neurons by ghrelin. Similarly, *in vivo* work has suggested that the histaminergic system is influenced by glucagon-like peptide-1 (GLP-1), as central GLP-1 infusion has been shown to increase histamine and histamine metabolite levels in the hypothalamus (154). The same study also indicated that the histaminergic system was required for the full anorexigenic effect of GLP-1, as inhibition of histamine synthesis (with α-FMH) attenuated the GLP-1 induced suppression of food intake (154). While there are descriptions of GLP-1 receptor (GLP-1R) expression in the TMN (155) and tuberal region (156), and GLP-1R agonists have been reported to activate (c-Fos) in the ventral region of the TMN (157) and the tuberal region (158), single cell sequencing fails to detect *Glp1r* mRNA in histaminergic neurons (153). Interestingly, the LH, a region strongly innervating the TMN, has been shown to express *Glp1r* mRNA (155, 156) and is involved in mediating some of the anorexigenic effects of GLP-1 (159). Thus, any influence of GLP-1 on the histaminergic neurons may be indirect via neurons of the LH.

The pancreatic hormone insulin may also have a role in regulating histaminergic neuron function. One study demonstrated that a very small percentage of histaminergic neurons displayed c-Fos expression following insulin-induced hypoglycemia (160). Further work would be required to delineate whether histaminergic neuron activation in these conditions was mediated by the hypoglycemia or insulin itself. However, histaminergic neurons have been shown to express the insulin receptor (153). Another metabolically relevant neuropeptide known to target the histaminergic neurons is orexin (also known as hypocretin). Orexin is a potent stimulator of feeding and the neurons synthesizing this orexigenic neuropeptide are located in the LH (161, 162). Importantly, histaminergic neurons express the orexin receptor type 2 (OxR2/Hcrt2) (153, 163) and are excited by orexin-A (164, 165). Orexin actions on histaminergic neurons have largely been demonstrated to influence arousal control (165, 166). However, it is important to note that the orexin neurons also co-express glutamate and can signal to the downstream histaminergic neurons via glutamatergic currents (22, 167). Moreover, the glutamatergic tone at histaminergic neurons, arising from the LH, has been linked to the modulation of food intake (147). While it is interesting to speculate about the different functional consequences of orexin neuronal transmission to histaminergic neurons, delineating such multifunctionality remains understudied. Overall, it appears that the ability of histaminergic neurons to detect, and interact with, metabolic signals occur via indirect (presynaptic) mechanisms, or via actions downstream of the histaminergic neurons themselves, i.e. on neurons expressing the histamine receptors.

## Medications regulating body weight via the histaminergic system

### Psychiatric medications for the treatment of schizophrenia

Supporting the importance of histamine receptors in the regulation of energy balance, antipsychotic medications that interact with the histaminergic system are associated with clinically significant weight gain (168, 169). Notably, the atypical antipsychotics with the largest weight gain profiles, olanzapine and clozapine, also display high affinities for the H1R (170–173). Atypical antipsychotics act to antagonize histamine’s endogenous actions at the H1R, which may partially explain the increased food intake seen with these medications (174–177). While the exact mechanisms underlying atypical antipsychotic-induced weight gain remain somewhat elusive, these medications have been shown to downregulate hypothalamic expression of the H1R (178). In addition, atypical antipsychotics have been shown to increase orexigenic neuropeptide Y (NPY) expression and activate the cellular energy sensor AMP-activated protein kinase (AMPK) in the hypothalamus, effects that are dependent on functional H1Rs (171, 179). Moreover, combination therapies including betahistine, a H1R agonist/H3R antagonist, have been shown to reduce weight gain in people treated with olanzapine (180). Although the histaminergic system is not the only transmitter system implicated in atypical antipsychotic-induced weight gain, strong evidence suggests its ability to influence food intake, and sensitivity to these medications, plays a contributing role.

### Therapeutic potential for the treatment of obesity

Following the cloning of the H3R in 1999 (58), numerous ligands were developed to manipulate the function of the receptor, and the H3R was subsequently proposed as a potential therapeutic target for the treatment of obesity (81, 116, 181, 182). In addition to food intake effects of H3R antagonists/inverse agonists, as discussed in this review, pre-clinical work demonstrated that these compounds also improve metabolic health and are associated with decreased body weight and fat mass, improved glucose homeostasis, and increased insulin sensitivity (108–111). These properties saw multiple pharmaceutical companies including Novo Nordisk, Abbott Laboratories, and Gliatech pursue H3R ligands for the treatment of obesity (183). While there was a brief surge in interest in these compounds for their metabolic effects, few ligands transitioned from the pre-clinical stage. Abbott laboratories H3R antagonist (A-331440) was found to have the potential for genotoxic effects which prohibited its further development as an anti-obesity therapeutic (184). Contradictory results were obtained between ligands with some studies failing to demonstrate consistent effects on food intake and anti-obesity properties (81, 185). Additionally, human trials with betahistine, a H1R agonist/H3R antagonist, failed to identify any striking weight loss effects in obese women (186), or on food intake when presented a buffet meal following a single day of betahistine treatment (187). Differences in ligand affinity for the H3R found between species may also contribute to some discrepancies observed among rodent and human studies (63, 188). Even though the pharmaceutical industry appears to have largely withdrawn its interest in pursuing the H3R as an anti-obesity target (189), work endures to optimize H3R ligands and explore their potential to influence food intake and body weight, and H3R antagonists/inverse agonists continue to be proposed for the treatment of obesity (190, 191).

## Considerations and future directions

The central histaminergic system has received considerable interest for its ability to regulate energy balance, however, many unanswered questions remain. Generally, histamine is considered an anorexigenic substance, as activation of H1Rs decrease food intake, effects that are believed to be mediated through actions in the PVH and VMH (12, 13). However, these hypothalamic nuclei consist of multiple cell types, and the chemical phenotype or identity of the cells mediating H1R agonism-induced suppression of food intake remain unidentified. Moreover, the view of histamine as an anorexigenic compound seems somewhat contradictory given that feeding occurs during waking hours when histaminergic neurons are active and histamine levels are highest (192–196). It appears that the picture is more complex and likely involves numerous interactions, some of which have yet to be uncovered.

Pharmacological and genetic knockout studies have provided important insights into the functioning of the histaminergic system, but the expression of histamine receptors in both the brain and periphery, and effects of the H3R on multiple neurotransmitter systems, likely complicate the interpretation of some of these findings. The field now requires the ability to manipulate individual histamine receptors in a cell-type specific way (e.g., histamine receptor floxed mice) to further delineate the precise actions of histamine in different nuclei and different cell types, and to overcome some of the inherent limitations of global knockout models.

Evidence also continues to emerge that the histaminergic neurons are heterogeneous. Differences have been demonstrated in their basal electrophysiological properties, transcriptional makeup, and their response to various pharmacological agents (163, 197–199). Such heterogeneity combined with multiple histamine receptors, differentially expressed within the hypothalamus and in multiple cell types, contributes to the complexity of the histaminergic system and highlights multiple ways histamine may serve to influence neuronal activity and food intake. Additionally, histamine itself has been proposed to function more like a neuromodulator or neuropeptide than a classical “neurotransmitter” (1, 200). Histaminergic neurons rarely form close synaptic contacts (201, 202), preventing their potential for traditional fast synaptic signaling to clearly defined post-synaptic targets. Rather, histaminergic neurons are believed to communicate via volume transmission, with histamine being released non-synaptically, allowing it to have longer lasting actions, and modulate neurotransmission at extra synaptic sites similar to other monoamines (203). The ability of histamine to signal in this fashion raises the potential for histaminergic neurons to “prime” other neurons’ responsiveness to additional incoming (metabolic) stimuli during waking hours when histaminergic tone is highest. However, future studies will be required to address such a possibility.

## Conclusion

In summary, histamine functions as a neuromodulator in the brain and contributes to the central regulation of energy homeostasis. Its effects on food intake largely depend on the histamine receptor subtype activated, with agonism of H1Rs being anorexigenic and agonism of H3Rs causing an orexigenic response. These important metabolic effects of HR activation contribute towards the weight gain side effects of some common medications and have seen HR ligands proposed as anti-obesity therapeutics. The histaminergic system has also been demonstrated to interact with key metabolic signals in the brain. It is clear that the histaminergic system has a powerful ability to influence food intake. Now we must turn our attention to elucidating the exact mechanisms by which it does so and the circumstances in which histaminergic signaling may contribute to an altered homeostatic drive to eat.

## Author contributions

All authors contributed to the article and approved the submitted version.
